# *trans*-Resveratrol Ameliorates Stress-Induced Irritable Bowel Syndrome-Like Behaviors by Regulation of Brain-Gut Axis

**DOI:** 10.3389/fphar.2018.00631

**Published:** 2018-06-15

**Authors:** Ying Xu, Su-Ying Cui, Quan Ma, Jing Shi, Ying Yu, Jian-Xin Li, Liang Zheng, Yi Zhang, Jian-Min Si, Ying-Cong Yu

**Affiliations:** ^1^Department of Gastroenterology, Wenzhou No. 3 Clinical Institute Affiliated to Wenzhou Medical University, Wenzhou People's Hospital, Wenzhou, China; ^2^Departments of Pharmaceutical Sciences, School of Pharmacy and Pharmaceutical Sciences, State University of New York at Buffalo, Buffalo, NY, United States; ^3^Department of Pharmacology, Peking University, School of Basic Medical Science, Beijing, China; ^4^School of Pharmacy, Hangzhou Medical College, Hangzhou, China; ^5^Institute of Gastroenterology, Zhejiang University, Hangzhou, China

**Keywords:** irritable bowel syndrome, depression, anxiety, *trans*-Resveratrol, phosphodiesterases 4A

## Abstract

**Background:** Irritable bowel syndrome (IBS) is a functional disorder characterized by abdominal pain and abnormalities in defecation associated with psychiatric disorders such as depression and anxiety due to the dysfunction of brain-gut axis. This study aims to determine whether *trans*-Resveratrol affects chronic-acute combined stress (CACS)-induced IBS-like symptoms including depression, anxiety and intestinal dysfunction.

**Methods:** ICR male mice were exposed to the CACS for 3 weeks. *trans*-Resveratrol were administrated daily (2.5, 5, and 10 mg/kg, i.g.) 30 min before CACS. Behavioral tests were performed to evaluate the treatment effects of *trans*-Resveratrol on IBS. Hippocampus tissues were collected and processed Golgi staining and immuno-blot analysis. Ileum and colon tissues were collected and processed Hematoxylin and Eosin staining and immuno-blot analysis.

**Results:** Administration with *trans*-Resveratrol before CACS for 3 weeks significantly reversed CACS-induced depression- and anxiety-like behaviors and intestinal dysfunction in mice, which implied a crucial role of *trans*-Resveratrol in treatment of IBS-like disorder. Furthermore, *trans*-Resveratrol improved hippocampal neuronal remodeling, protected ileal and colonic epithelial barrier structure against CACS insults. The further study suggested that *trans*-Resveratrol normalized phosphodiesterases 4A (PDE4A) expression and CREB-BDNF signaling that were disturbed by CACS. The increased pCREB and BDNF expression in the hippocampus were found, while decreased pCREB and BDNF levels were observed after treatment with *trans*-Resveratrol.

**Conclusions:** The dual effects of *trans*-Resveratrol on stress-induced psychiatric and intestinal dysfunction may be related to normalization of PDE4A expression and subsequent pCREB-BDNF signaling in the hippocampus, ileum and colon.

## Introduction

Irritable bowel syndrome (IBS) is characterized by a functional disorder with abdominal pain and defecation, affecting up to 7–21% of the population worldwide (Chey et al., 2015). Over the past several decades, a large body of research supports that the negative emotion such as depression or anxiety plays a major role in gut functioning due to the bidirectional communications between gut and brain, namely, the brain-gut axis (Dinan and Cryan, 2017; Pellissier and Bonaz, 2017). Antidepressants have become a widespread treatment option for IBS patients, because of their effects on pain perception, mood, and motility (Dekel et al., 2013; Chey et al., 2015). However, the application of classical antipsychotic drugs for the treatment of IBS is very restricted as results of the serious adverse effects, i.e., vomiting and 2 weeks delayed efficacy. Therefore, it would be beneficial to the IBS patients if they were exposed to a drug that has dual effects on psychosis and intestinal dysfunction without adverse effects.

*trans*-Resveratrol (3,5,4′-trihydroxy-*trans*-stilbene) is a polyphenol extracted from *Polygonum cuspidatum*, the skin of red grapes and red berries. *trans*-Resveratrol has several biochemical actions and appears to involve a combination of anti-oxidant, anti-inflammatory, and neuroprotective activities by targeting various molecular such as brain-derived neurotrophic factor (BDNF) and phosphodiesterases (PDEs) (Chung, 2012; Park et al., 2012). Pharmacokinetic studies demonstrate *trans-*Resveratrol could be detectable both in plasma and brain, causing both peripheral and central effects (Sale et al., 2004; Frozza et al., 2010). Our and others' studies have demonstrated that *trans-*Resveratrol exhibited potential antidepressant and anxiolytic-like effects in various animal models (Damián et al., 2014; Ali et al., 2015; Ge et al., 2016; Xu et al., 2016) and increased BDNF protein levels in the hippocampus (Wang et al., 2016c). Furthermore, *trans-*Resveratrol was shown to protect intestinal barrier function against oxidative stress (Wang et al., 2016b). Based on these findings, it is possible that *trans-*Resveratrol might have dual effects on central nervous and peripheral disorders, i.e. psychiatric and intestinal dysfunction. Increasing evidence suggested that *trans*-Resveratrol exerts various kinds of pharmacology effects by competitive inhibition of cAMP-degrading PDEs (Chung, 2012; Park et al., 2012) followed by amplification of cAMP-mediated signal cascade and modulation of the special proteins such as BDNF (Wang et al., 2016a). Among the 11 members of the PDE family (PDE1-11), PDE4 is of particular interest in the IBS pathology due to its high expression in brain-gut axis (Barnette et al., 1993; Johansson et al., 2012). PDE4 inhibitors alleviate stress-induced depression-like behaviors by increasing immobility time in the forced swimming test and intestinal dysfunction by reducing defecation (Barnette et al., 1993; O'Donnell and Zhang, 2004; Barone et al., 2008; Wang et al., 2013, 2015; McGirr et al., 2016). These results imply that *trans-*Resveratrol may inhibit PDE4 subtypes and the downstream signaling, leading to effective treatment of IBS-like symptoms. Among the 4 subtypes of PDE4 (PDE4A-D), PDE4A is highly vulnerable to chronic stress, which confirmed by our unpublished data that suggested a dramatic increase in the mRNA level of PDE4A in the hippocampus following chronic stress. Therefore, the present study particularly focused on the role of PDE4A dependent signaling in the protective effects of *trans-*Resveratrol against IBS insults.

Considering the important role of PDE4 in the psychology and gastroenterology disorders and the inhibitory effects of *trans-*Resveratrol on PDE, we hypothesize that *trans-*Resveratrol may reverse IBS-related depression-, anxiety-like behaviors and intestinal dysfunction by regulation of brain-gut axis. The present study investigated the dual effects of *trans-*Resveratrol on central nervous and peripheral dysfunction induced by chronic-acute combined stress (CACS). The PDE4A mediated signals, such as PDE4A, pCREB and BDNF expression in the hippocampus, ileum and colon were also investigated.

## Materials and methods

### Animals

A total of 96 male ICR mice weighting about 30 g were used (Harlan, Indianapolis, IN). Mice were housed under standard laboratory conditions, with a 12 h light/dark cycle and had free access to food and water. They were allowed at least 1 h of habituation before any experiment was performed. All behavioral tests were carried out under “NIH Guide for the Care and Use of Laboratory Animals” (revised 2011) and were approved by the Institutional Animal Care and Use Committee of State University of New York at Buffalo and Wenzhou Medical University.

### Drugs and treatments

*trans*-Resveratrol, desipramine and diazepam were purchased from Sigma Chemical Co. (St. Louis MO, USA). Rolipram was provided by A.G. Scientific (San Diego, CA). *trans-*Resveratrol was prepared to suspension with 0.5% sodium carboxymethyl cellulose and administrated by gavage (i.g.) in the volume of 0.01 ml/g. Rolipram, desipramine and diazepam were separately dissolved in saline and were administrated by intraperitoneal injection (i.p) in the volume of 0.01 ml/g. Vehicle (0.5% sodium carboxymethyl cellulose, i.g.), *trans-*Resveratrol (2.5, 5.0, and 10.0 mg/kg, i.g.), rolipram (1.25 mg/kg, i.p.), desipramine (10 mg/kg, i.p.) or diazepam (0.5 mg/kg, i.p.) were administrated daily 30 min before the chronic acute combining stress (CACS) for 19 days. Rolipram, a PDE4 inhibitor, were used to evaluate the role of PDE4 in the pathogenesis of IBS. Desipramine is a classical anti-depressant and diazepam is a classical anxiolytic, which were used as positive controls to assess behavioral test validity. Behavioral tests were performed from day 20–23.

### Chronic-acute combined stress (CACS) model

In the CACS group, mice were exposed to the following conditions described previously (Xu et al., 2006; Yu et al., 2013) with minor modifications. Stress was administered twice per day over a period of 19 days. The sequence of stress was arranged in Table 1. Mice were subjected to behavior tests between 09:00 and 16:00 from day 20–23. Animals were sacrificed on day 23 to determined intestinal motility and the mouse whole brain, hippocampus, ileum and colon tissues were collected for morphological and immuno-blot analyses.

**Table 1:** Chronic-acute combined stress (CACS) procedure.

**Day**	**Stress**
1	Cold environment (4°C, 15 min) and food deprivation (4 h)
2	Cage tilting and water deprivation (4 h)
3	Bedding damp (2 h) and cold swim (12°C, 5 min)
4	Restraint (2 h) and tail pinch (5 min)
5	Food & water deprivation (6 h) and foot shock (3 min; 36 V)
6	Cold swim (12°C, 10 min) and overnight illumination (12 h)
7	Bedding damp (4 h) and tail pinch (10 min)
8	Restraint (3 h) and cold swim (12°C, 5 min)
9	Tail pinch (15 min) and overnight illumination (12 h)
10	Bedding damp (6 h) and foot shock (6 min; 36 V)
11	Restraint (4 h) and overnight illumination (12 h)
12	Cold swim (12°C, 10 min)
13	Overnight bedding damp (12 h) and tail pinch (20 min)
14	Restraint (6 h)
15	Cage tilting (6 h) and foot shock (10 min; 36 V)
16	Cold swim (12°C, 10 min) Food & water deprivation (6 h)
17	Overnight bedding damp (12 h) and tail pinch (30 min)
18	Cold environment (4°C, 20 min) and overnight illumination (12 h)
19	Restraint (6 h)

### Sucrose preference test

Mice were exposed to both 1% sucrose solution and double distilled water for 5-day regimen of sucrose preference test as previously described (Wang et al., 2015). Five-day regimen performed continuously throughout the CACS paradigm. Briefly, mice were individually housed and subjected to two identical bottles that were filled with double distilled water (water/water) on the first 2 days and with sucrose solution (sucrose/sucrose) on the next 2 days. On the fifth day, all the mice were given a free choice between two bottles (water/sucrose) for 12 h from 21:00 to 09:00. The position of bottles was changed in the middle of the test (03:00). The sucrose preference was calculated as the ratio of sucrose consumed to the total amount of liquid consumed (water and sucrose liquid). The sucrose preference test was performed on day 0 and 20.

### Forced swimming test

Mice were subjected a swimming-stress period for 6 min in a glass cylinder (20 cm diameter, 45 cm height) filled with water (23 ± 1°C; depth 30 cm) on day 21. Immobility time was judged to be immobile only with the small movements when it ceased struggling and remained floating motionless in the water and was recorded for the last 4 min. Forced swimming test was performed on day 21 (Yu et al., 2015).

### Open field test

Mice were individually placed in a black Plexiglas box (50 × 50 × 39 cm) with the box floor divided into nine identical squares in a dim room. The distance in the center area, time spent in the center area and velocities were recorded in a 5 min period. After each test, the box was cleaned with 75% ethanol solution to clear the potential clues from the previous test. Open field test was performed on day 21 before forced swimming test (Zhang et al., 2013).

### Elevated plus-maze

The elevated plus-maze apparatus including a central platform (5 × 5 cm), two open arms (30 × 5 cm) and two closed arms (30 × 5 × 15) that were located in a dim room. The elevated plus-maze apparatus was elevated 50 cm above the floor. In the elevated plus-maze test session, each mouse was placed in the central platform and allowed to freely explore for 5 min. The percentage of open arm entries was obtained as the number of open-arms entries divided by total open and closed arms entries. The percentage of time spent in open arms was obtained as time spent in the open arms divided by total time. Elevated plus-maze test was performed on day 22 (Zhang et al., 2017).

### Abdominal withdrawal reflex (AWR) test

In order to study visceral sensitivity to rectal distension, the visceral hyperalgesia to colorectal distention was assessed by AWR according to previous study with minor modifications (O'Mahony et al., 2012). A disposable silicon balloon-urethral catheter for pediatric use (6 Fr, Terumo, Tokyo, Japan) was used in this experiment. Mice were briefly anesthetized with isoflurane. The balloon was inserted into the rectum until the catheter was positioned to the anus (2 cm distal from the end of the balloon), then the catheter was fixed to the base of the tail to prevent detachment. After the mice were completely recovered from the anesthesia, they were placed to the transparent cage and allowed to acclimate for a minimum of 30 min before testing. Ascending-limit phasic distension (0.25, 0.35, 0.50, or 0.65 ml) was applied for 30 s every 4 min. In this experiment, the AWR was semiquantitatively scored as previously described (Al-Chaer et al., 2000). The AWR score was assigned as follows: 0 = no behavioral response to distension, 1 = brief head movements followed by immobility, 2 = contraction of abdominal muscle without lifting of abdomen, 3 = lifting of abdomen, 4 = body arching and lifting of pelvic structure. Rectal distension procedure was performed on day 22 after elevated plus-maze test.

### Intestinal motility assay

In order to measure the motility of the intestinal, the intestinal motility assay was performed according to previously studies (Zhou et al., 2013). One day after behavioral tests, mice were administrated with methylene blue-labeled 10% dextrose solution (Sigma-Aldrich) by gavage (1 g/kg, i.g.) after periods of food deprivation (6 h). Mice were euthanized 30 min after injection of the blue dye. Intestinal transit was measured from the pylorus to the most distal point of migration of the blue dye, and then the total length of the small intestine was recorded. The ratio of the dye migration distance/total intestinal length was calculated. Intestinal motility assay was performed on day 23.

### Rapid golgi staining in hippocampus

Mice brains were quickly taken out and processed according to the protocols of the rapid Golgi staining kit (FD NeuroTechnologies, Ellicott City, MD). Briefly, serial sections (100 μm, 1 in 9 series) were obtained through the whole hippocampus (−1.4 to −2.4 mm from the bregma) on a freezing microtome (Paxinos and Franklin, 2001). Brain sections were dehydrated in alcohol, cleared in xylene, and mounted in neutral balsam. For morphological analysis of hippocampal neurons, 5 pyramidal neurons from each mouse (4 mice/group, 20 brain sections from each group) were calculated from area CA1 of the hippocampus. A camera lucida drawing tube attached to an Olympus microscope BX51 (Olympus, Tokyo, Japan) was applied to find selected neurons (400 ×) for computerized image analysis. The center of the soma was as the reference dot, the total dendritic length and the number of dendrites were measured every 50 μm. Meanwhile, the spine density (per 10 μm distances) was quantified (Shankaranarayana Rao et al., 2001; Vyas et al., 2002).

### HE staining in ileum and colon

Ileum and colon samples from the mice were fixed with 4% paraformaldehyde and embedded in paraffin and were cut into 5 μm-thick sections. To evaluate the overall enteral inflammation state, the series sections (1 in 5 series, 5 sections from each mouse, 4 mice/group, 20 sections for each group) were stained with haematoxylin and eosin (HE) following standard protocols and then observed with microscope (Olympus, Tokyo, Japan). A histopathological score was based on the mucosal architecture (0–3, normal to extensive damage), cellular infiltrate (0–3, no infiltrate to transmural infiltration), muscle thickening (0–3, normal to extensive thickening), crypt abscesses (0 absent. 1 present) and goblet cell depletion (0 absent, 1 present). (Dieleman et al., 1998).

### Immunoblot analysis

Mouse brain (8 mice/group) was lysed with RIPA buffer (Sigma Chemical Co., St. Louis MO, USA) containing protease and phosphatase inhibitors, and then centrifuged at 12,000 rpm for 20 min at 4°C. 30–90 μg of the protein samples were denatured with 5 × loading buffer at the temperature of 90–95°C and then separated on 10% SDS-PAGE gels. Proteins from the gels were transferred to polyvinylidene difluoride (PVDF) membranes, blocked with blocking buffer (PBS containing 3% BSA and 0.1% sodium azide) and incubated with primary antibodies, i.e., PDE4A (abcam, Cambridge, MA), phospho-CREB at Ser 133 (abcam, Cambridge, MA), CREB (abcam, Cambridge, MA), BDNF (abcam, Cambridge, MA), β-actin (Santa Cruz Biotechnology, Dallas, Texas), overnight at 4°C. After that, the PVDF membranes were incubated with the secondary horseradish peroxidase-conjugated antibody (1:5,000) at 25°C for 1 h. Labeled protein bands were detected using enhanced chemiluminescence (ECL) method and quantified using ImageJ software (National Institutes of Health, USA).

### Statistical analyses

All values were expressed as mean ± standard error of the mean (SEM). For multiple comparisons, data were analyzed statistically using one-way analysis of variance (ANOVA), followed by a post hoc Dunnett's test. For two groups comparisons, data were analyzed statistically using *t*-test. Difference with *P* < 0.05 was considered as statistically significant.

## Results

### *trans-*resveratrol exhibited antidepressant- and anxiolytic-like effects

**Figure 1:**
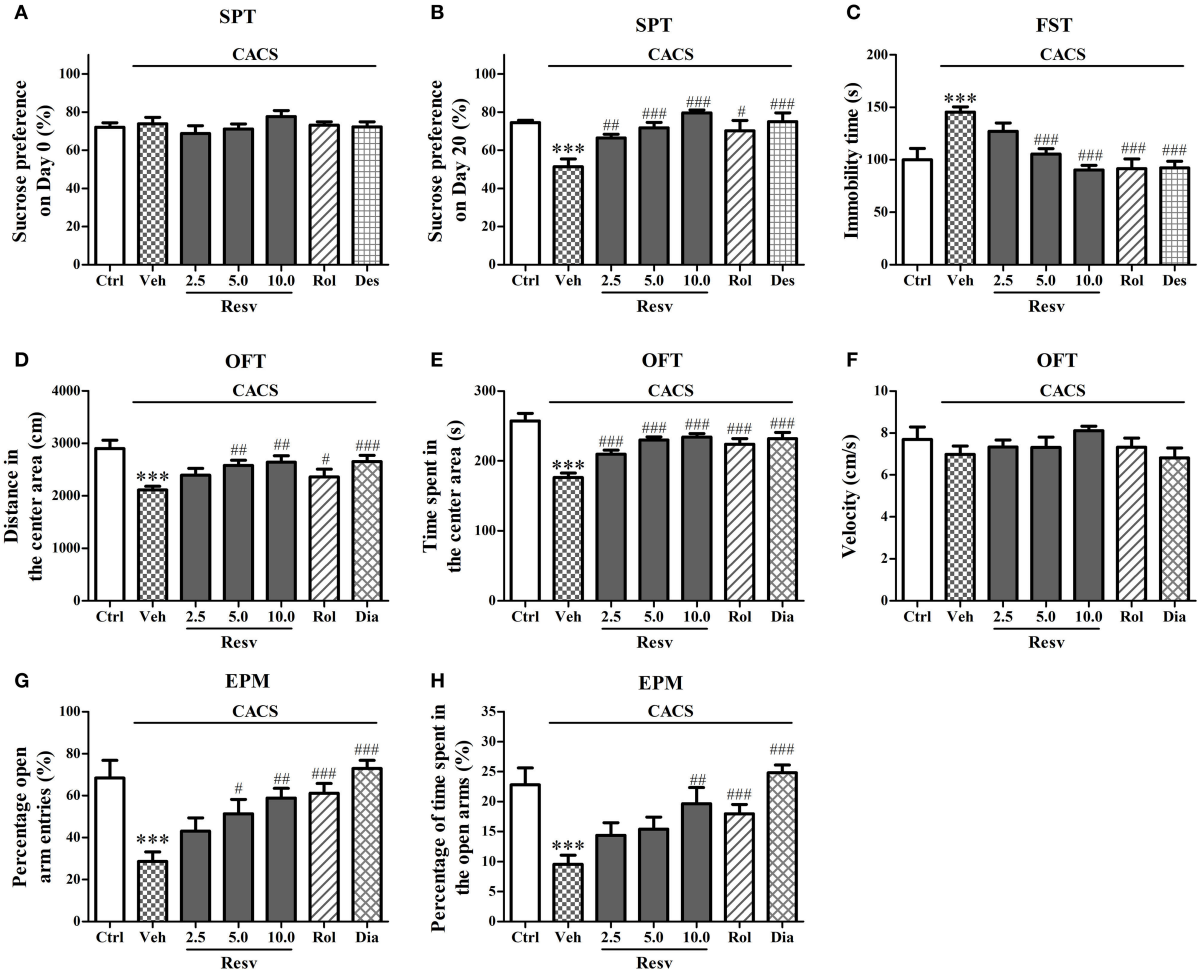
The antidepressant- and anxiolytic-like effects of *trans*-Resveratrol on mice exposed to chronic-acute combined stress (CACS). Mice were exposed to stress (subjected to CACS) and daily treated with vehicle (Veh), *trans-*Resveratrol (Resv, 2.5, 5.0 and 10.0 mg/kg, i.g.), rolipram (Rol, 1.25 mg/kg, i.p.) or desipramine (Des, 0.5 mg/kg, i.p.) in SPT and FST, or diazepam (Dia, 10 mg/kg, i.p.) in OFT and EPM 30 min before stress for 3 weeks. The sucrose preference ratio was measured in sucrose preference test (SPT) on day 0 and day 20 (**A,B**, respectively). Immobility time was measured in forced swimming test (FST) on day 21 **(C)**. The distance in the center area, time spent in the center area and velocities were measured in the open field test (OFT) on day 21 **(D–F)**. The percentage of open arm entries and the percentage of time spent in open arms were measured in the elevated plus-maze (EPM) test on day 22 **(G,H)**. Results are expressed as mean ± SEM (*n* = 12). ^***^P < 0.001 vs. control group (Ctrl), ^#^*P* < 0.05, ^##^*P* < 0.01, and ^###^*P* < 0.001 vs. CACS + Veh group.

The potential antidepressant-like effects of *trans-*Resveratrol were evaluated in sucrose preference and the forced swimming tests. The sucrose preference ratio was not significant across the groups on the day 0 (Figure 1A). However, the CACS-treated group showed significant decrease in sucrose preference ratio on the day 20 (*P* < 0.001, Figure 1B). Treatment with *trans-*Resveratrol (Resv, 2.5, 5, or 10 mg/kg, i.g.) for 3 weeks before CACS significantly increased the sucrose preference ratio on the day 20, as compared to vehicle-treated CACS group [*F*_(3, 44)_ = 18.06, *P* < 0.001, Figure 1B]. Treatment with rolipram (PDE4 inhibitor, the positive drug, Rol, 1.25 mg/kg, i.p.) or desipramine (Des, 0.5 mg/kg, i.p.) also significantly increased the sucrose preference ratio on the day 20 (*P* < 0.05; *P* < 0.001; Figure 1B).

In the forced swimming test, mice subjected to the CACS procedure for 3 weeks showed longer immobility time than that of control group (*P* < 0.001, Figure 1C). Mice treated with *trans-*Resveratrol (5 and 10 mg/kg) before CACS for 3 weeks decreased the immobility time than those of CACS group [*F*_(3, 44)_ = 17.23, *P* < 0.001, Figure 1C]. Rolipram or desipramine was also shown to decrease the immobility time when compared to vehicle-treated CACS group (*P's* < 0.001; Figure 1C). These results suggested that *trans-*Resveratrol exhibited anti-depressant like effects in the CACS mouse model.

The potential anxiolytic-like effects of *trans-*Resveratrol were evaluated in two anxiety tests, open field and elevated plus-maze tests. As shown in Figures 1D–H, both the distance (*P* < 0.001) and the time spent (*P* < 0.001) in the center area were clearly shorter in mice subjected to the CACS procedure for 3 weeks in the open field test. Treatment with *trans-*Resveratrol before CACS for 3 weeks significantly ameliorated CACS-induced behavioral abnormalities by increasing the distance [*F*_(3, 44)_ = 4.95, *P* < 0.01] and the time spent [*F*_(3, 44)_ = 23.77, *P* < 0.001] in the center area. Treatment with rolipram before CACS for 3 weeks increased the time spent in the center area when compared to vehicle-treated CACS group (*P* < 0.001). The classical anxiolytic drug diazepam (Dia, 10 mg/kg, i.p.) also increased the distance (*P* < 0.001) and the time spent (*P* < 0.001) in the center area as compared to vehicle-treated CACS group. Statistical differences of the velocity were not observed across the groups as shown in Figure 1F.

In the elevated plus-maze test, 3 weeks CACS exposure significantly reduced the percentage of open arm entries (*P* < 0.001, Figure 1G) and percentage of time spent in the open arms (*P* < 0.001, Figure 1H) when compared to those of controls. Treatment with *trans-*Resveratrol before CACS for 3 weeks significantly reversed CACS-induced anxiogenic-like effects, as evidenced by increasing percentage of open arm entries [*F*_(3, 44)_ = 5.21, *P* < 0.01, Figure 1G] and percentage of time spent in open arms [*F*_(3, 44)_ = 3.16, *P* < 0.05, Figure 1H]. Rolipram and diazepam also significantly increased percentage of open arm entries and percentage of time spent in the open arms (*P's* < 0.001, Figures 1G,H). These results suggested that *trans-*Resveratrol produced anxiolytic-like effects in the mouse model of CACS.

### *trans-*resveratrol ameliorated intestinal dysfunction

**Figure 2:**
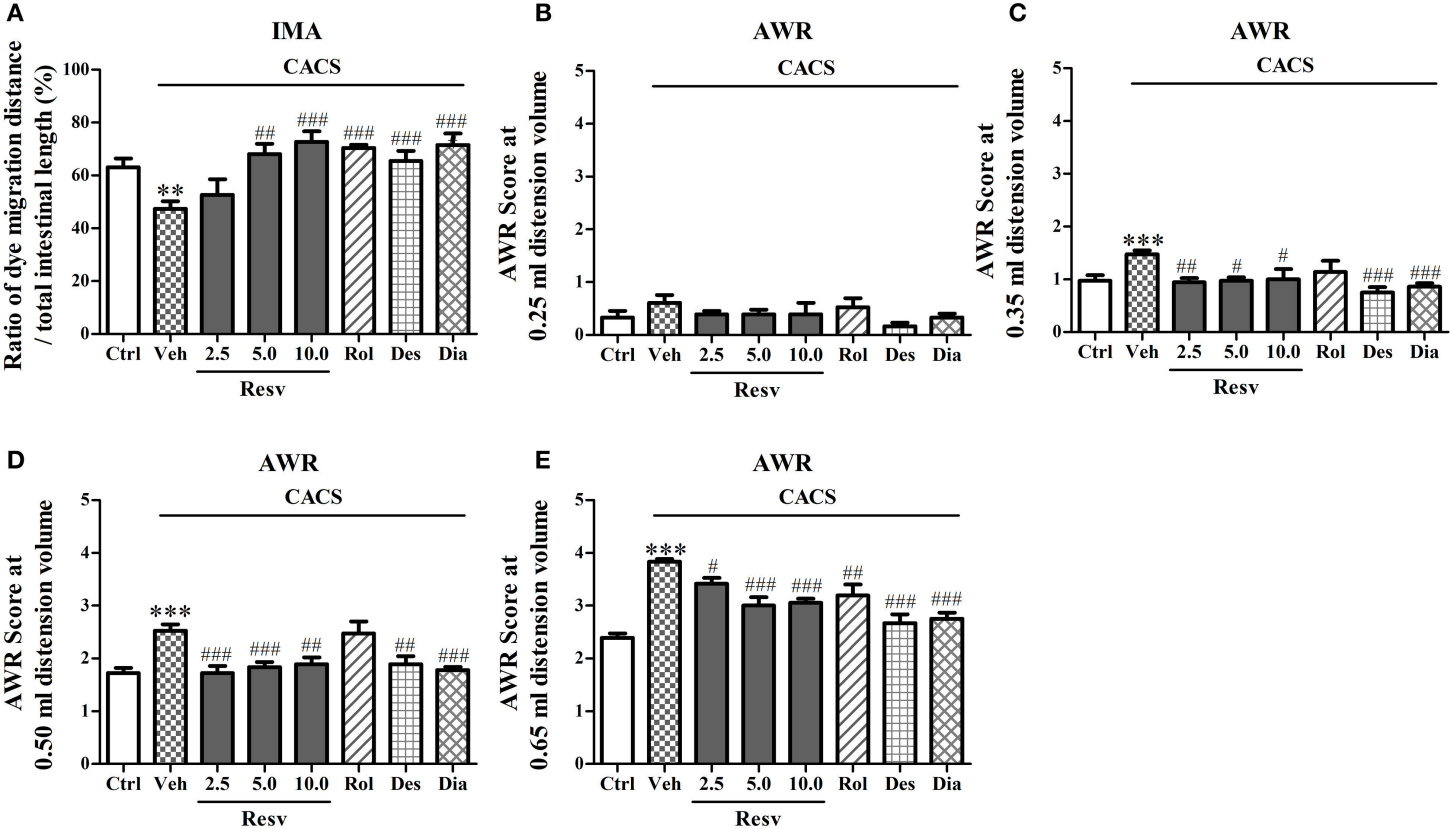
The peripheral effects of *trans-*Resveratrol on mice exposed to CACS. Mice were exposed to stress (subjected to CACS) and daily treated with vehicle (Veh), *trans-*Resveratrol (Resv, 2.5, 5.0, and 10.0 mg/kg, i.g.), rolipram (Rol, 1.25 mg/kg, i.p.), desipramine (Des, 0.5 mg/kg, i.p.) or diazepam (Dia, 10 mg/kg, i.p.) 30 min before stress for 3 weeks. **(A)** The ratio of the dye migration distance to the total intestinal length was measured in intestinal motility assay (IMA) test on day 23. **(B–E)** The abdominal withdrawal reflex (AWR) scores at 0.25, 0.35, 0.50, and 0.65 ml distension volumes were measured in AWR test on day 22. Results are expressed as mean ± SEM (*n* = 12). ^**^P < 0.01 and ^***^P < 0.001 vs. Ctrl group, ^#^*P* < 0.05, ^##^*P* < 0.01, and ^###^*P* < 0.001 vs. CACS + Veh group.

The potential protective effects of *trans-*Resveratrol on intestinal function were evaluated in two tests, intestinal motility assay and abdominal withdrawal reflex test. 3 weeks CACS exposure significantly decreased gastrointestinal motility (*P* < 0.01, Figure 2A). Treatment with *trans-*Resveratrol (2.5, 5, and 10 mg/kg, i.g.) before CACS for 3 weeks significantly rescued CACS-induced abnormality of intestinal motility, as shown by the increased ratio of dye migration distance to total intestinal length, as compared to vehicle-treated control group [*F*_(3, 44)_ = 7.85, *P* < 0.001, Figure 2A]. Rolipram, desipramine, and diazepam also significantly suppressed CACS-induced gastrointestinal hypomotility (*P* < 0.001, Figure 2A).

Abdominal withdrawal reflex (AWR) test is introduced as a semi-quantitative method to gauge involuntary motor reflex in response to visceral pain (Al-Chaer et al., 2000). It involves a supraspinal loop and is quantified by assigning a numerical score to the graded contractions of the abdominal muscles. Statistical differences in the AWR scores at 0.25 ml distension volume were not observed across the groups after 3 weeks of CACS exposure (Figure 2B). While significantly increased AWR scores at 0.35, 0.50, and 0.65 ml distension volumes (*P* < 0.001, Figures 2C–E) were observed. Treatment with *trans-*Resveratrol before CACS for 3 weeks rescued CACS-induced visceral hypersensitivity by decreasing AWR scores at 0.35 ml [*F*_(3, 44)_ = 4.72, *P* < 0.01], 0.50 ml [*F*_(3, 44)_ = 8.92, *P* < 0.001] and 0.65 ml [*F*_(3, 44)_ = 12.84, *P* < 0.001] distension volumes, as compared to vehicle-treated CACS group (Figures 2C–E). Rolipram also decreased AWR scores at 0.65 ml (*P* < 0.01) distension volumes, as compared to vehicle-treated CACS group (Figure 2E). Desipramine and diazepam decreased AWR scores at 0.35 ml, 0.50 and 0.65 ml distension volumes (*P* < 0.01 or < 0.001, Figures 2C–E). The results suggested that *trans-*Resveratrol exhibited anti-IBS-like effects in mouse model of IBS disorders.

### *trans-*resveratrol improved neuronal remodeling in the hippocampus

**Figure 3:**
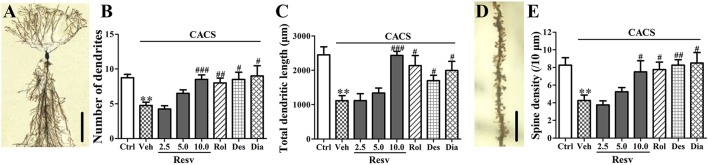
The potential protective effects of *trans-*Resveratrol on hippocampal neuronal remodeling against CACS. Mice were exposed to stress (subjected to CACS) and daily treated with vehicle (Veh), *trans-*Resveratrol (Resv, 2.5, 5.0, and 10.0 mg/kg, i.g.), rolipram (Rol, 1.25 mg/kg, i.p.), desipramine (Des, 0.5 mg/kg, i.p.), or diazepam (Dia, 10 mg/kg, i.p.) 30 min before stress for 3 weeks. **(A)** Photomicrographs of representative Golgi stained hippocampal CA1 pyramidal neuron (scale bar = 50 μm). **(B,C)** Number of dendrites and total dendritic length were quantified. **(D)** Photomicrographs of representative Golgi stained hippocampal CA1 pyramidal neuron's spine (scale bar = 5 μm). **(E)** Spine density (10 μm distances) was quantified. Results are expressed as mean ± SEM (*n* = 4). ^**^P < 0.01 vs. Ctrl group, ^#^*P* < 0.05, ^##^*P* < 0.01, and ^###^*P* < 0.001 vs. CACS + Veh group.

Three weeks CACS exposure caused a significant decrease in the number of dendrites, the total dendritic length and spine density in the hippocampal CA1 pyramidal neurons, as compared to vehicle-treated control animals (*P* < 0.01, Figure 3). Treatment with *trans-*Resveratrol before CACS significantly reversed CACS-induced atrophy in the hippocampal CA1 pyramidal neurons by increasing number of dendrites [*F*_(3, 12)_ = 13.19, *P* < 0.001], total dendritic length [*F*_(3, 12)_ = 16.47, *P* < 0.001] and spine density [*F*_(3, 12)_ = 4.54, *P* < 0.05, Figure 3]. Treatment with rolipram, desipramine or diazepam before CACS also increased the number of dendrites, the total dendritic length and spine density in the hippocampal CA1 pyramidal neurons, as compared to those of vehicle-treated CACS animals (*P's* < 0.05 or *P's* < 0.01, Figure 3). These results suggested that *trans-*Resveratrol protected hippocampal neuronal remodeling against CACS insults.

### *trans*-resveratrol exhibited anti-inflammatory effects on the ileum and colon

**Figure 4:**
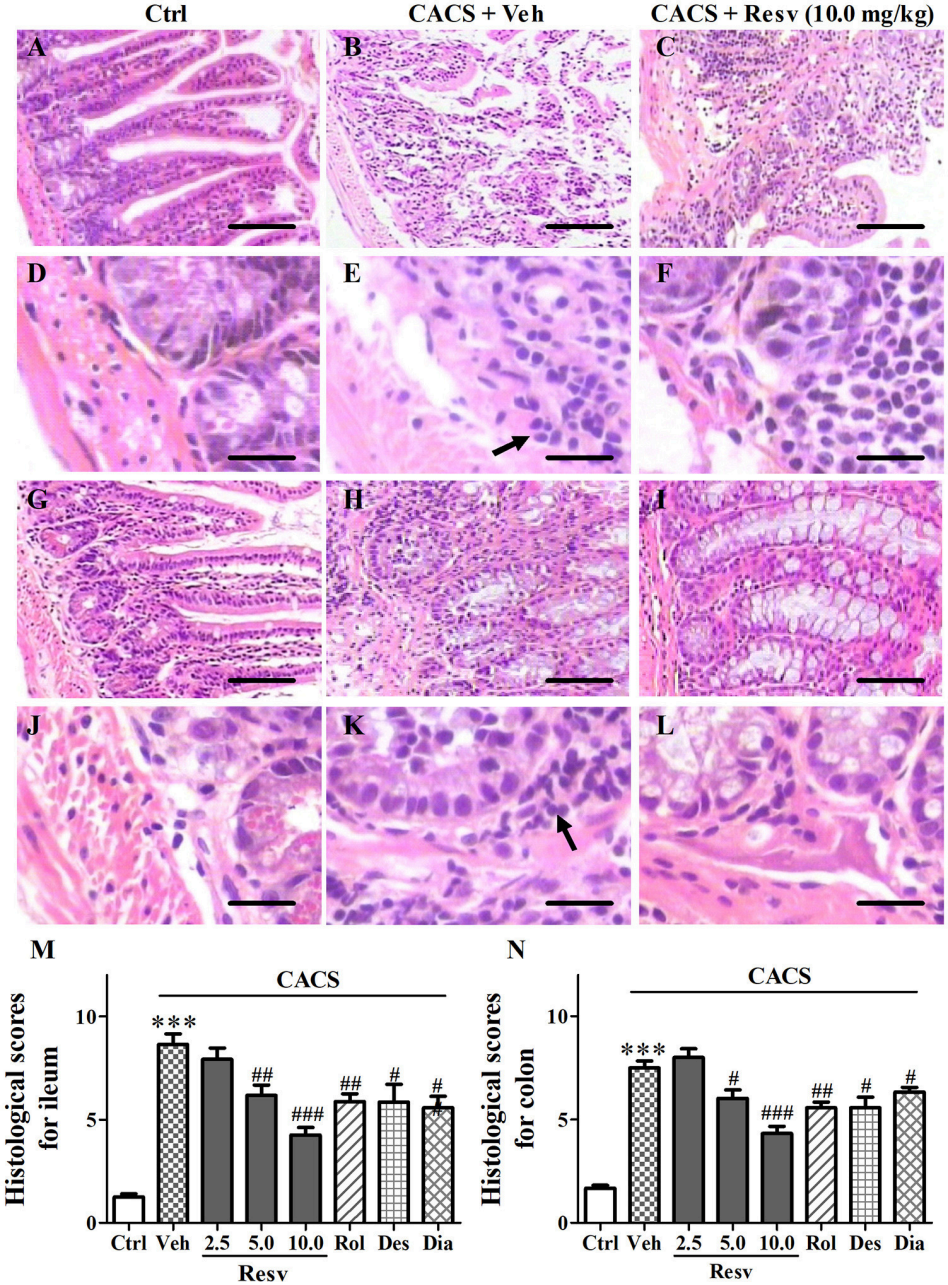
The intestinal structural changes after treatment with *trans-*Resveratrol in CACS mice. Mice were exposed to stress (subjected to CACS) and daily treated with vehicle (Veh), *trans-*Resveratrol (Resv, 2.5, 5.0 and 10.0 mg/kg, i.g.), rolipram (Rol, 1.25 mg/kg, i.p.), desipramine (Des, 0.5 mg/kg, i.p.) or diazepam (Dia, 10 mg/kg, i.p.) 30 min before stress for 3 weeks. **(A–F)** Photomicrographs of representative HE staining in the ileum. **(G–L)** Photomicrographs of representative HE staining in the colon. Immune cellular infiltrate is indicated by arrow. Scale bar = 100 μm **(A–C, G–I)** and 10 μm **(D–F, J–L)**. **(M,N)** Histopathological scores representing the severity of inflammation were quantified in the ileum and colon. Results are expressed as mean ± SEM (*n* = 4). ^***^P < 0.001 vs. Ctrl group, ^#^*P* < 0.05, ^##^*P* < 0.01, and ^###^*P* < 0.001 vs. CACS + Veh group.

Macroscopic assessment of the ileal and colonic mucosa including hyperemia, ulcers, bowel wall thickening, structure and adhesion, was evaluated by histological score. The higher score indicates the more serious inflammatory damage. Histological scores were significantly higher in mice subjected to CACS for 3 weeks than those of control groups, which demonstrated that the significant inflammatory damage on ileum and colon in CACS mice (*P's* < 0.001, Figure 4). Chronic *trans*-Resveratrol treatment dose-dependently reduced histological scores in the ileum [*F*_(3, 12)_ = 16.25, *P* < 0.001] and colon [*F*_(3, 12)_ = 18.60, *P* < 0.001], as compared to vehicle-treated CACS groups. However, *trans*-Resveratrol did not completely reverse CACS-induced ileal and colonic damage. Rolipram, desipramine, or diazepam also reduced histological scores in the ileum and colon, as compared to vehicle-treated CACS groups (*P* < 0.05 or < 0.01, Figures 4M,N). These results suggested that mice subjected to 3 weeks CACS suffered ileal and colonic mucosa inflammatory damage. *trans-*Resveratrol produced anti-inflammatory effects on CACS induced IBS model.

### *trans-*resveratrol normalized PDE4A, pCREB, and BDNF protein levels in the hippocampus

**Figure 5:**
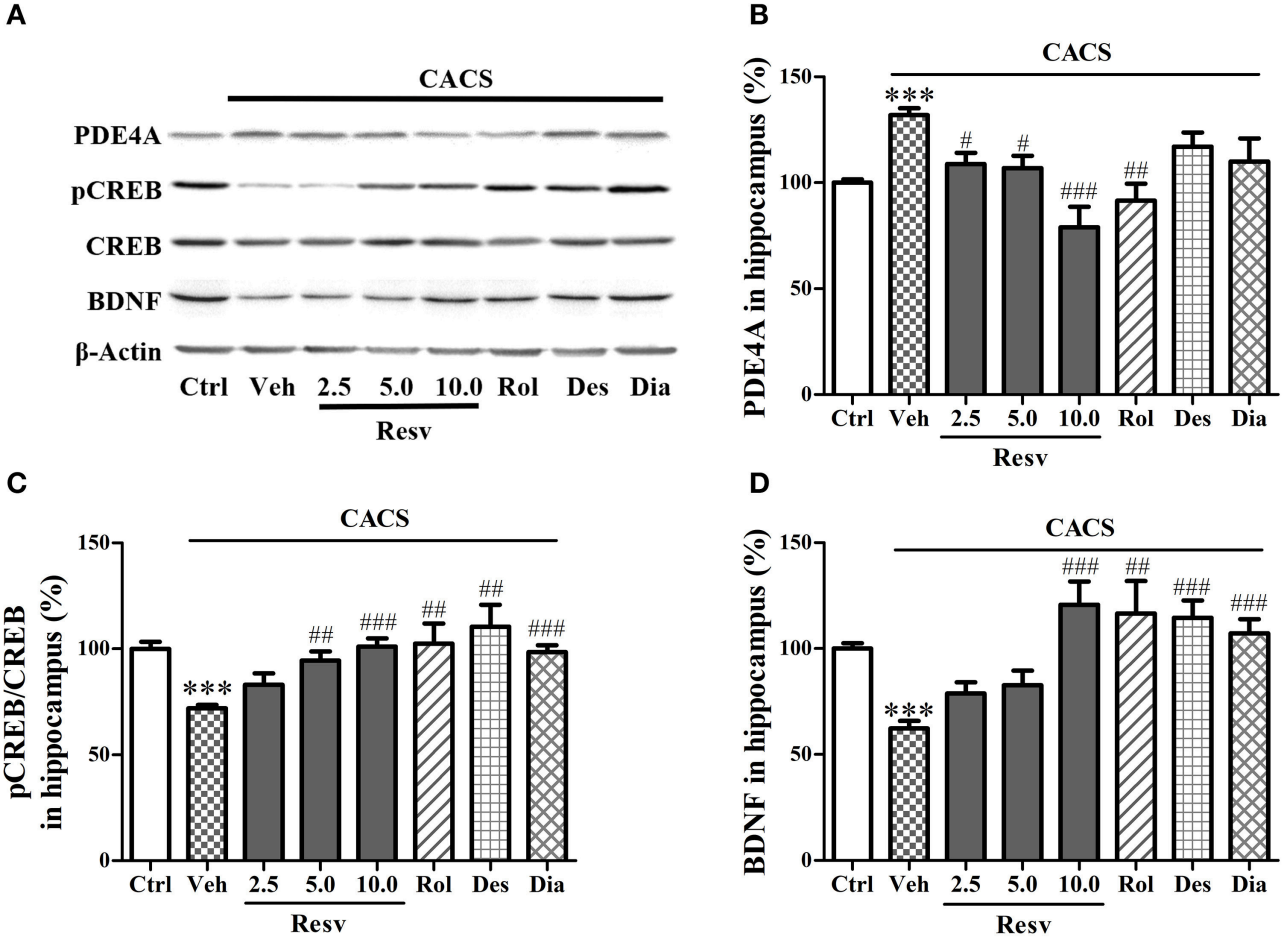
Immuno-blot analyses for pCREB and BDNF expression in the hippocampus. Mice were exposed to stress (subjected to CACS) and daily treated with vehicle (Veh), *trans-*Resveratrol (Resv, 2.5, 5.0, and 10.0 mg/kg, i.g.), rolipram (Rol, 1.25 mg/kg, i.p.), desipramine (Des, 0.5 mg/kg, i.p.) or diazepam (Dia, 10 mg/kg, i.p.) 30 min before stress for 3 weeks. **(A)** Photomicrographs of representative immune-blotting bands; **(B–D)** PDE4A expression, the ratio of pCREB to total CREB and BDNF protein levels in the hippocampus were measured. Results are expressed as mean ± SEM (*n* = 8). ^***^P < 0.001 vs. Ctrl group, ^#^*P* < 0.05, ^##^*P* < 0.01 and ^###^*P* < 0.001 vs. CACS + Veh group.

Immuno-blot analyses in the hippocampus showed that 3 weeks CACS exposure significantly increased the PDE4A protein level (*P* < 0.001), decreased the ratio of pCREB to total CREB (pCREB/CREB) and BDNF levels (*P's* < 0.001), as compared to those of control groups (Figures 5A–D). Treatment with *trans-*Resveratrol before CACS for 3 weeks significantly decreased PDE4A protein levels at 2.5, 5, and 10 mg/kg [*F*_(3, 28)_ = 11.28, *P* < 0.001], increased the ratio of pCREB to total CREB [*F*_(3, 28)_ = 10.01, *P* < 0.001] and BDNF expression [*F*_(3, 28)_ = 11.54, *P* < 0.001]. Rolipram also decreased PDE4A level, increased pCREB/CREB and BDNF protein levels in the hippocampus, as compared to vehicle-treated CACS groups (*P* < 0.01 or < 0.001). Two positive drugs desipramine and diazepam had similar effects on pCREB/CREB level and BDNF (*P* < 0.001 or < 0.01). These results suggested that *trans-*Resveratrol reversed the effects of CACS on PDE4A, pCREB/CREB, and BDNF expression in the hippocampus.

### *trans-*resveratrol normalized PDE4A, pCREB, and BDNF protein level in the ileum and colon

**Figure 6:**
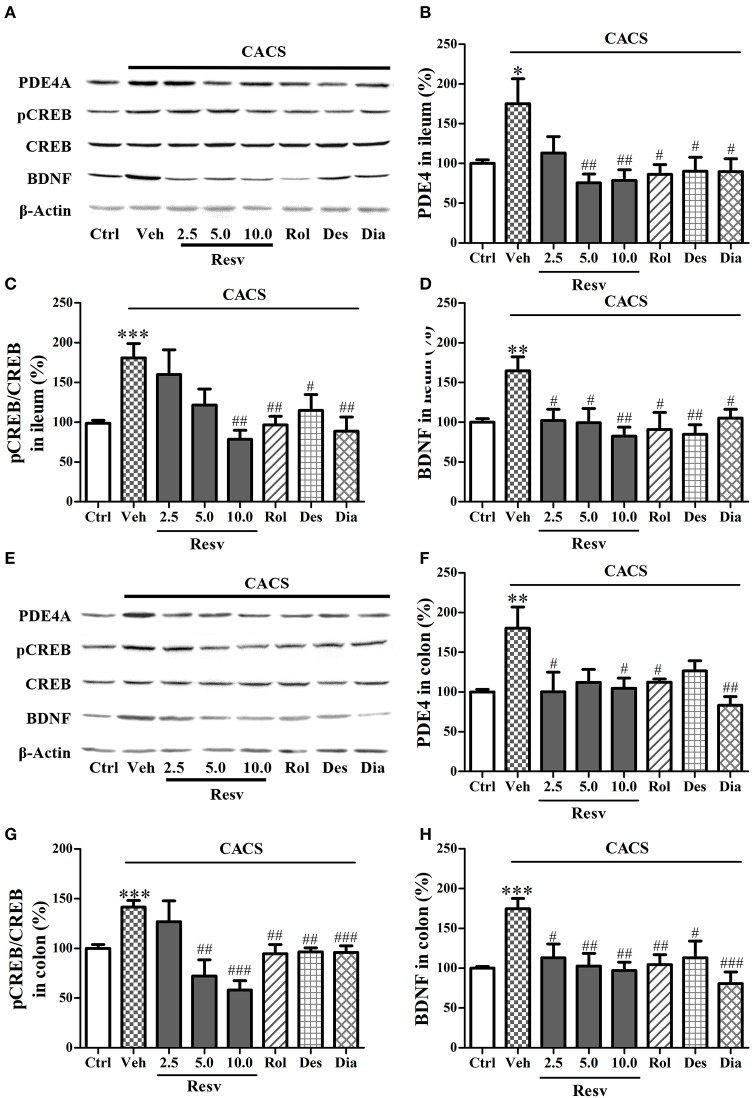
Immuno-blot analyses for pCREB and BDNF expression in the ileum and colon. Mice were exposed to the stress (subjected to CACS) and daily treated with vehicle (Veh), *trans-*Resveratrol (Resv, 2.5, 5.0, and 10.0 mg/kg, i.g.), rolipram (Rol, 1.25 mg/kg, i.p.), desipramine (Des, 0.5 mg/kg, i.p.) or diazepam (Dia, 10 mg/kg, i.p.) 30 min before stress for 3 weeks. **(A)** Photomicrographs of representative immune-blotting bands; **(B–D)** PDE4A expression, the ratio of pCREB to total CREB and BDNF protein level in the ileum were measured. **(E)** Photomicrographs of representative immune-blotting bands. **(F–H)** PDE4A, the ratio of pCREB to total CREB and BDNF levels were measured in the colon. Results are expressed as mean ± SEM (*n* = 8). ^*^P < 0.05, ^**^P < 0.01, and ^***^P < 0.001 vs. Ctrl group, ^#^*P* < 0.05, ^##^*P* < 0.01, and ^###^*P* < 0.001 vs. CACS + Veh group.

As shown in Figures 6A–D, CACS exposure for 3 weeks significantly increased PDE4A (*P* < 0.05), pCREB/CREB (*P* < 0.001), and BDNF protein expression (*P* < 0.01) in the ileum, when compared to control groups. Treatment with *trans-*Resveratrol reversed the effects of CACS on ileum by decreasing PDE4A expression at 5 and 10 mg/kg (*P's* < 0.01), pCREB/CREB at 10 mg/kg (*P* < 0.01), and BDNF protein levels at all three doses [*F*_(3, 28)_ = 5.49, *P* < 0.01]. Positive drugs including rolipram, desipramine, and diazepam also decreased PDE4A expression, pCREB/CREB, and BDNF expression (*P* < 0.05 or < 0.01) in the ileum.

The data from immuno-blot analyses in the colon also showed that CACS significantly increased PDE4A, pCREB/CREB, and BDNF protein levels when compared to control groups (*P* < 0.01 or < 0.001, Figures 6E–H). Treatment with *trans-*Resveratrol reversed these proteins expression in colon by decreasing PDE4A at 5 and 10 mg/kg (*P* < 0.05), pCREB/CREB at 5 and 10 mg/kg (*P* < 0.001), and BDNF expression at 2.5, 5, and 10 mg/kg [*F*_(3, 28)_ = 6.19, *P* < 0.01]. Positive drugs also decreased PDE4A, pCREB/CREB, and BDNF expression, as compared to vehicle-treated CACS groups (*P* < 0.001 or < 0.01 or < 0.05). These results suggested that *trans-*Resveratrol ameliorated CACS-induced abnormalities of PDE4A expression and CREB-BDNF signaling in the ileum and colon.

## Discussion

The present study suggested that mice exposed to the CACS for 3 weeks induced a series of abnormalities both in affective and somatic dysfunction, i.e., depression- and anxiety-like behaviors, gastrointestinal hypomotility and hypersensitivity, which mimic IBS symptoms in patients. Treatment with *trans-*Resveratrol produced dual effects both in central nervous and intestinal systems. Behavioral studies suggested that *trans-*Resveratrol not only exhibited antidepressant- and anxiolytic- like effects but also ameliorated gastrointestinal hypomotility and hypersensitivity. Furthermore, histological studies showed that *trans-*Resveratrol treatment significantly increased the number of dendrites, dendritic length and spine density of hippocampal neurons and partially decreased the inflammatory damage caused by CACS in the ileum and colon. These *trans-*Resveratrol-induced behavioral and morphological changes seemed to differentially affect signal molecules in brain-gut axis, as evidenced by the fact that *trans-*Resveratrol decreased PDE4A expression in the hippocampus, ileum and colon, increased pCREB and BDNF protein levels in the hippocampus; while pCREB and BDNF levels were decreased in the ileum and colon after treatment with *trans-*Resveratrol. These dual effects of *trans-*Resveratrol on central nervous and peripheral systems demonstrated that *trans-*Resveratrol ameliorates CACS-induced IBS-like symptoms by differentially regulating histological and molecular abnormalities in the brain-gut axis.

Chronic and acute stress animal models are extensively utilized to unravel the biological mechanisms underlying affective and somatic disorders. Animals subjected to chronic acute combining stress result in emotional disorders, abnormal intestinal motility and visceral hypersensitivity (Mineur et al., 2007; Ping et al., 2014; Yu et al., 2015; Moloney et al., 2016). In this study, the CACS procedure was used to mimic the symptoms of IBS including depression, anxiety and bowel disorders. The results suggested that *trans-*Resveratrol significantly ameliorated CACS-induced depression- and anxiety-like behaviors, which were confirmed by a series of behavioral tests including sucrose preference, forced swimming, open field and elevated plus-maze tests. Indeed, the antidepressant and anxiolytic-like effects of *trans-*Resveratrol have been reported in our previous studies (Xu et al., 2010), but the effects of *trans-*Resveratrol on IBS by regulation of both affective and somatic disorders remained unknown. The previous study suggested that *trans-*Resveratrol produces an antidepressant-like effect by reversing corticosterone-induced decreased sucrose consumption in sucrose preference test and decreasing immobility time in forced swimming test (Ali et al., 2015). Another study supports the above finding, which suggests that *trans-*Resveratrol alleviates both depression- and anxiety-like behaviors in the subclinical hypothyroidism rats (Ge et al., 2016). The present study extended the previous studies and found for the first time that *trans-*Resveratrol not only ameliorated CACS-induced depression- and anxiety-like behaviors, but also significantly reversed CACS-induced gastrointestinal hypomotility and visceral hypersensitivity. The effects of *trans-*Resveratrol on intestinal system were consistent with previous study that demonstrated that *trans-*Resveratrol ameliorated acute small intestinal inflammation by down-regulating immune response and prevented bacterial translocation *via* maintaining intestinal barrier function (Bereswill et al., 2010). Similar results showed that *trans-*Resveratrol protected intestinal barrier function against oxidative stress (Wang et al., 2016b). The present results, together with findings from other studies, demonstrated that *trans-*Resveratrol significantly improved CACS-induced both psychiatric and gastrointestinal dysfunction, implying the potential therapeutic value of *trans-*Resveratrol on IBS.

The intestinal tract is highly susceptible to stress, where barrier dysfunction develops rapidly and can be long lasting. A defective mucosal barrier has been implicated in the development of intestinal inflammation and hyperalgesia (Gareau et al., 2008). In the present study, we observed that the CACS exposure caused ileal and colonic epithelial barrier structure dysfunction and inflammation. Although the complex gut-brain interactions involved in the pathophysiology of stress-related psychiatric comorbidity of IBS is not well known, preclinical and clinical researches indicate that the hippocampus, which is known for its role in the regulation of stress-induced emotional behavior and visceral hypersensitivity, might be a key component (Zhang et al., 2016). The previous study demonstrated that the induction of visceral pain by colorectal distention increased the release of hippocampal noradrenaline in animal models (Saito et al., 2002). Another study provided preliminary evidence for the presence of abnormal hypofunction of hippocampal glutamatergic neurotransmission in IBS patients as a result of the chronic pain (Niddam et al., 2011). The abnormalities of neurotransmitters in the hippocampus could affect neuroplasticity, which might be associated with the pathophysiology of depression and anxiety in IBS patients. In the present study, we found that the neuronal remodeling in the hippocampus was significantly damaged by the CACS exposure in mouse model of IBS disorder. Interestingly, the administration of *trans-*Resveratrol protected ileal and colonic epithelial barrier structure, as well as remodeling of hippocampal neurons against CACS. The dual protective effects of *trans-*Resveratrol on both intestinal mucosa and hippocampal neurons might be great advantages in the IBS treatment. It is possible that *trans-*Resveratrol could not only relieve abdominal pain by directly suppressing gastrointestinal inflammation, but also abolish psychiatric comorbidity by enhancing hippocampal neuronal remodeling. Future study should be clarified the involvement of other brain regions especially limbic system (e.g., amygdala, prefrontal cortex) in the treatment effects of *trans-*Resveratrol on IBS.

*trans-*Resveratrol is thought to exert part of its actions by inhibition of PDEs (Chung, 2012; Park et al., 2012). Inhibition of PDE4 increases cyclic adenosine monophosphate (cAMP), which activates cAMP response element-binding protein (CREB) and eventually enhances special gene transcription such as BDNF (Fujimaki et al., 2000). In the present study, we investigated whether PDE4A, CREB, and BDNF were involved in the dual effects of *trans-*Resveratrol on both hippocampal neurons and intestinal mucosa in CACS-induced mouse model of IBS disorders. Anatomical and histological studies showed that PDE4 is highly expressed in the brain regions most relevant to depression and anxiety, i.e., hippocampus, as well as in the digestive tract, i.e., ileum and colon (Barnette et al., 1993; Johansson et al., 2012). Upregulation of PDE4 induced by CACS may inhibit CREB-BDNF-mediated neurogenesis in the hippocampus, which is associated with the pathogenesis of depression and anxiety (O'Donnell and Zhang, 2004; Xu et al., 2011; Wang et al., 2014; Zhang et al., 2017). In addition, PDE4 appears to be important in modulating contractile activity of colonic smooth muscle and PDE4 inhibition is effective in reducing rodent stress-induced defecation (Barnette et al., 1993; Barone et al., 2008). Clinical and preclinical studies suggested that BDNF acts on the intestinal sensory nerve endings, may promote their growth and synapse formation and alter epithelial barrier, which lead to visceral hypersensitivity in IBS patient and CACS-induced mouse model (Yu et al., 2012, 2015, 2017). Among the PDE4 family, PDE4A subtype is highly vulnerable to chronic stress. We analyzed the correlations between behavioral phenotype and biochemical markers (Supplementary Figures 1, 2). These results indicate PDE4A and BDNF in brain-gut axis might be involved in CACS-induced IBS-like behaviors. Present study showed that *trans-*Resveratrol reversed CACS-induced increase in PDE4A in the hippocampus, ileum and colon, which might contribute to the IBS treatment. The interesting findings of our study were that the opposite effects of *trans-*Resveratrol on pCREB and BDNF expression in the hippocampus and intestines, i.e., increased pCREB, BDNF expression in the hippocampus and decreased pCREB, BDNF levels in the ileum and colon. The upregulation of pCREB and BDNF in the hippocampus might be contribute to ameliorating psychiatric disorders; while downregulation of pCREB and BDNF in the intestines might be contribute to recovering the intestinal dysfunction. We speculate that *trans-*Resveratrol may decrease PDE4A expression and consequently enhance cAMP-medicated pCREB-BDNF signaling pathway in the hippocampus and decrease pCREB and BDNF expression in the intestine as a regulator for negative feedback mechanism in the intestine. However, the future work is necessary to decipher the causal relationship between PDE4A dependent CREB-BDNF signaling and the effects of *trans*-Resveratrol.

In summary, CACS procedure stimulates a series of symptoms of IBS including depression, anxiety and intestinal dysfunction due to morphological and biochemical alterations in IBS mouse model. *trans-*Resveratrol exhibited dual effects on affective and somatic symptoms, i.e. not only relieved CACS-induced depression- and anxiety- like behaviors, but also ameliorated intestinal dysfunction. The underlying mechanism may be related to normalization of PDE4A expression and CREB-BDNF signaling both in the central nervous and peripheral systems. *trans-*Resveratrol may be served as a novel multi-target agent for treatment of IBS via its dual effects on affective and somatic disorders.

## Author contributions

YX, S-YC and Y-CY designed and prepared the manuscript. QM, JS, YY, J-XL, and LZ performed the experiments. J-MS and YZ analyzed the data.

### Conflict of interest statement

The authors declare that the research was conducted in the absence of any commercial or financial relationships that could be construed as a potential conflict of interest.
