# Supplementary material for: trans-Resveratrol Ameliorates Stress-Induced Irritable Bowel Syndrome-Like Behaviors by Regulation of Brain-Gut Axis
Source: Front Pharmacol. 2018 Jun 15;9:631. doi: 10.3389/fphar.2018.00631 (PMC6013570; doi:10.3389/fphar.2018.00631)
Supplement: Supplementary file 1 [file Data_Sheet_1.docx]

Supplementary Material

***trans*-Resveratrol** **ameliorates irritable bowel syndrome-like behaviors by regulation of brain-gut axis**

Ying Xu^1, 2^ *, Su-Ying Cui^2, 3^*, Quan Ma^2^ *, Jing Shi^4^, Jian-Xin Li^1^, Ying Yu^1^, Liang Zheng^1^, Yi Zhang^2^ Han-Ting Zhang^5^, Ying-Cong Yu^1 #^

^1^ Department of Gastroenterology, Wenzhou No. 3 Clinical Institute of Wenzhou Medical University, Wenzhou people’s hospital, Wenzhou, Zhejiang Province 325000, China

^2^ Departments of Pharmaceutical Sciences, School of Pharmacy and Pharmaceutical Sciences, State University of New York at Buffalo, Buffalo, NY, 14214, USA

^3^ Department of pharmacology, Peking University, School of Basic Medical Science, 38 Xueyuan Road, Beijing, 100191, China

^4^ School of Pharmacy, Hangzhou Medical College, Hangzhou, Zhejiang Province 310053, China

^5^ Departments of Behavioral Medicine & Psychiatry and Physiology, Pharmacology & Neuroscience, Rockefeller Neurosciences Institute, West Virginia University Health Sciences Center, Morgantown, WV, 26506, USA

^*^ Authors contributed equally to this work.

^#^ Correspondence: Ying-Cong Yu

E-mail: [yingcongyu123@163.com](mailto:yingcongyu123@gmail.com)

Tel:86-577-88059910


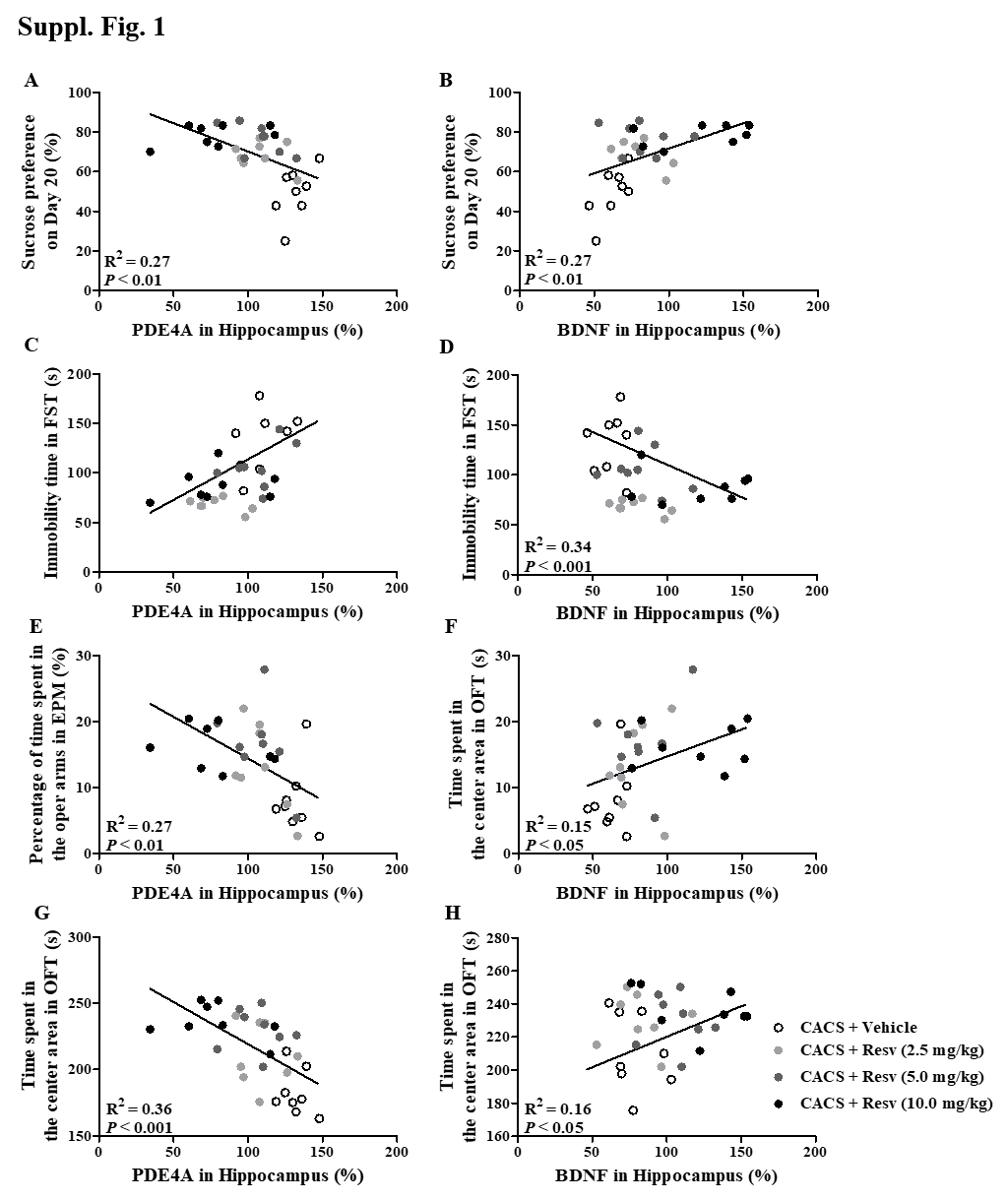


**Suppl. Fig. 1.** The correlations between depression-/anxiety-like behaviors and PDE4A/BDNF expression in hippocampus. Mice were exposed to chronic-acute combined stress (CACS) and daily treated with vehicle (Veh), trans-Resveratrol (Resv, 2.5, 5.0 and 10.0 mg/kg, i.g.). Depression-like behaviors were evaluated in sucrose preference (A and B) and the forced swimming tests (FST, C and D). Anxiety-like behaviors were evaluated in elevated plus-maze test (EPM, E and F) and open field test (OFT, G and H). (Pearson’s correlation analysis, n=8)


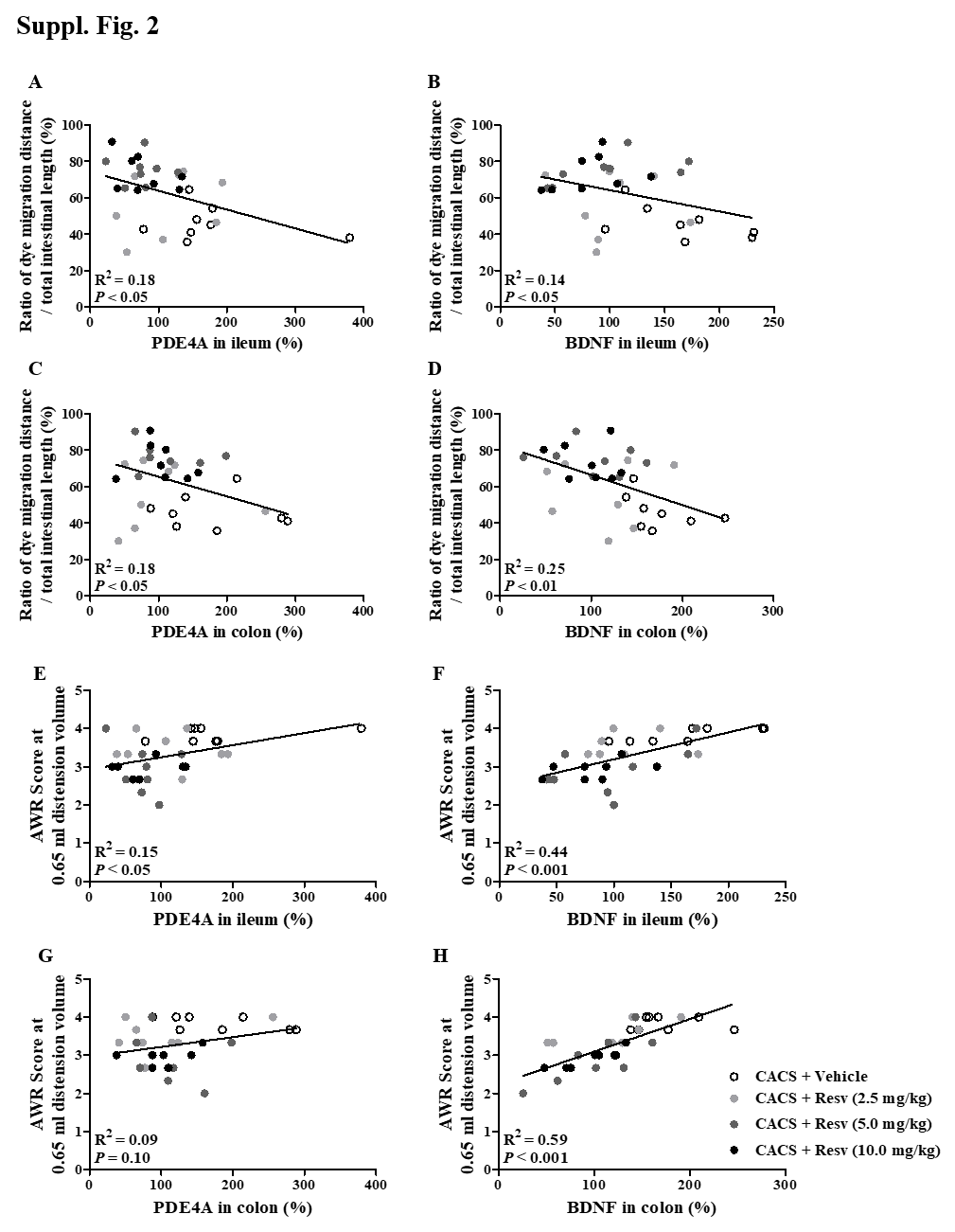


**Suppl. Fig. 2.** The correlations between intestinal dysfunctions and PDE4A/BDNF expression in ileum and colon. Mice were exposed to chronic-acute combined stress (CACS) and daily treated with vehicle (Veh), trans-Resveratrol (Resv, 2.5, 5.0 and 10.0 mg/kg, i.g.). Intestinal motility assay (A-D) and abdominal withdrawal reflex test (AWR, E-H) were performed to evaluate intestinal dysfunctions. (Pearson’s correlation analysis, n=8)
